# Left ventricular assist device implantation in advanced kidney disease

**DOI:** 10.1016/j.jhlto.2025.100422

**Published:** 2025-10-29

**Authors:** Benjamin N. French, Anudeep S. Nakirikanti, Ian Kusher, Kris Wittersheim, J. David Vega, Mani Daneshmand, Divya Gupta

**Affiliations:** a Emory University School of Medicine, Atlanta, GA; b Emory University Department of Cardiology, Atlanta, GA; c Emory University Division of Cardiothoracic Surgery, Atlanta, GA

**Keywords:** Left ventricular assist device (LVAD), HeartMate 3, Chronic kidney disease (CKD), Renal replacement therapy (RRT), Advanced heart failure, Survival outcomes

## Abstract

**Background:**

Advanced chronic kidney disease (CKD) is often considered a contraindication to left ventricular assist device (LVAD) implantation, yet limited data exist on outcomes in the era of the HeartMate 3 device.

**Methods:**

We retrospectively reviewed 193 patients who underwent HeartMate 3 LVAD implantation at a tertiary care academic medical center between July 2016 and July 2023. Patients were stratified by preoperative estimated glomerular filtration rate (eGFR) ≥30 mL/min/1.73 m² (control, *n* = 154) vs <30 (advanced kidney disease, *n* = 39). Outcomes included survival and the need for renal replacement therapy (RRT).

**Results:**

At 30 days post-implantation, survival was lower in advanced kidney disease patients compared with controls (84.6% vs 94.2%, *p* = 0.04). However, survival at 1 year (79.5% vs 79.9%, *p* = 0.87) and 2 years (73.9% vs 68.7%, *p* = 0.60) did not differ significantly. Post-implantation RRT was required in 30.3% of advanced kidney disease patients and 18.2% of controls (*p* = 0.18).

**Conclusions:**

Although early postoperative mortality is higher, patients with advanced CKD undergoing HeartMate 3 LVAD implantation achieve comparable 1- and 2-year survival to controls. These findings suggest LVAD implantation may be a reasonable option in carefully selected patients with advanced kidney disease.

## Background

Left ventricular assist devices (LVADs) are a lifesaving therapy for patients with severe heart failure as both a bridge to heart transplantation and as lifetime (destination) therapy. The only currently commercially available model is the HeartMate 3, which features an intrathoracic, fully magnetically levitated centrifugal-flow pump.^1^ The purpose of this new centrifugal-flow design is to reduce pump thrombosis and clot formation: major causes of mortality in older devices.^2^

Patients with heart failure often suffer from kidney disease, either chronic kidney disease (CKD) or acute cardiorenal syndrome when in a decompensated state. CKD stage 4 (defined as a glomerular filtration rate of 15-29) is a general contraindication to LVAD implantation, while end-stage renal disease is considered a hard contraindication at many centers.^3^ Existing data suggests that patients with CKD 4 and 5 can achieve similar mortality outcomes compared to controls^4^ but are at greater risk for progression to complete renal failure.^4^ However, evidence is limited, and at many institutions, advanced kidney disease is a limiting factor to LVAD therapy. Furthermore, LVAD technology continues to improve, and minimal studies have examined outcomes solely in patients who receive the newest HeartMate 3 device.

## Methods

### Study population

At our institution, 193 patients received a HeartMate 3 LVAD and were enrolled in our study between July 1st, 2016, and July 1st, 2023. Eight patients received a heart transplant and were censored at the time of transplantation. One patient left our system to continue their care in another part of the country and was censored at that time. The remaining patients were followed until July 1st, 2023, or until their time of death. Patient demographics are summarized in Table 1.

**Table 1:** Study Population Demographics. Six patients were on Renal Replacement Therapy at the time of implantation and were assigned an eGFR of 14 (highest possible eGFR to classify as Stage 5 CKD).

Characteristics	Total	Control (eGFR > 30)	Advanced kidney disease (eGFR < 30)	*p*-value
*N*	193	154	39	
eGFR	51.0 (±25.6)	58.8 (±22.6)	20.1 (±5.6)	2.2e-16*
Age	50.9 (±12.7)	51.0 (±12.7)	50.6 (±12.6)	0.98
Female, *n* (%)	59 (30.6%)	46 (29.9%)	13 (33.3%)	0.82
INTERMACS profile	2.2 (±0.6)	2.3 (±0.6)	1.8 (±0.6)	4.9e-5*
BMI	29.9 (±8.4)	30.0 (±8.6)	29.3 (±7.9)	0.90
Smoking history, *n* (%)	87 (45.1%)	72 (46.8%)	15 (38.5%)	0.43
Diabetes	74 (38.3%)	63 (40.9%)	11 (28.2%)	0.20
Hypertension	179 (92.7%)	142 (92.2%)	37 (94.9%)	0.82
Hemoglobin (g/dL)	10.1 (±2.0)	10.3 (±2.0)	9.3 (±2.0)	2.5e-3*
Total bilirubin (mg/dL)	1.7 (±1.9)	1.5 (±1.5)	2.3 (±3.0)	9.5e-3*

CKD, chronic kidney disease; eGFR, estimated glomerular filtration rate.

*Denotes a *p*-value < 0.05.

Patients with an estimated glomerular filtration rate (eGFR) less than 30 (henceforth described as “advanced kidney disease”) had mostly similar demographics to controls, save for a few categories. Most notably, advanced kidney disease patients tended to have a lower INTERMACS profile (1.8 vs 2.3) at the time of implantation. This finding is expected, as these patients demonstrated renal failure or near-failure on top of their coexisting heart failure, with several requiring dialysis in the days leading to implantation.

### Study design

This was a retrospective cohort study conducted via review of patient charts. Need for renal replacement was defined by the use of renal replacement therapy (RRT) at any time between implantation and the study endpoint. This study was approved by our institutional review board.

### Study groups

Patients were classified into cohorts based on their eGFR on the morning of surgery. eGFR was calculated based on the CKD-EPI formula.^5^ Patients with preop eGFR of 30 or greater were classified as our control group. Those with an eGFR of 29 or less were classified as advanced kidney disease. Of these patients, 10 were classified as end-stage renal disease: four patients had an eGFR less than 15, four patients required continuous renal replacement therapy (CRRT) prior to implantation, and 2 patients were chronically dialysis-dependent. In total, the control group consisted of 154 patients, and the advanced kidney disease group consisted of 39 patients.

### Statistical analysis

We used R version 4.3.1 and the survival^6^ and ggsurvfit^7^ packages to conduct analyses on patient mortality and to generate Kaplan-Meier curves. As our mortality data involved censored patients, we used log-rank testing to conduct between-subgroup significance testing for mortality.

For all other analyses, we used Mann-Whitney *U* testing to compare continuous variables and the chi-square test to compare categorical variables. Bar graphs were generated with the ggplot2^8^ package.

## Results

### Survival

**Figure 1:**
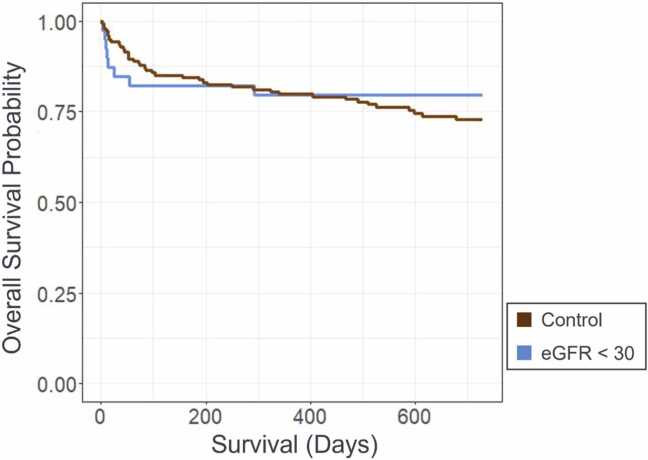
2-year survival in advanced kidney disease patients vs controls.

Our average follow-up period was 2.2 years. Survival data is described in Figure 1 and Table 2. We found a significant decrease in survival at 30 days post-implantation for advanced kidney disease patients; however, this difference in survival is no longer seen at the 1-year mark and beyond. At one and 2 years, we find no significant difference in survival between groups.

**Table 2:** Survival Data

	Total	Control (eGFR > 30)	Advanced kidney disease (eGFR < 30)	*p*-value
*N*	193	154	39	
30-day Survival	178/193 (92.2%)	145/154 (94.2%)	33/39 (84.6%)	0.04*
1-year Survival	154/193 (79.8%)	123/154 (79.9%)	31/39 (79.5%)	0.87
2-year Survival	95/137 (69.3%)	79/115 (68.7%)	17/23 (73.9%)	0.60

eGFR, estimated glomerular filtration rate.

*Denotes a *p*-value < 0.05.

Since some patients received an LVAD less than 2 years before the study endpoint, these patients were not included in the 2-year survival, hence the smaller denominators noted in that section of Table 2.

### Need for RRT

**Figure 2:**
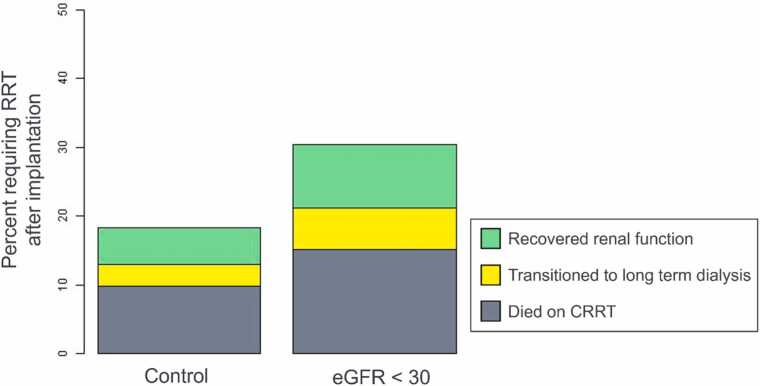
RRT requirements after LVAD implantation.

We describe new RRT requirements in Figure 2 and Table 3. We observe a trend toward significance (*p* = 0.18) but no significant difference in new RRT requirements between study groups.

**Table 3:** Renal Replacement Therapy Requirements After Implantation

	Total	Control (eGFR > 30)	Advanced Kidney Disease (eGFR < 30)	*p*-value
*N*	193	154	39	
Required RRT after implantation	38/193 (19.7%)	28/154 (18.2%)	10/33 (30.3%)	0.18
Recovered renal function	11/38 (28.9%)	8/28 (28.6%)	3/10 (33.3%)	
Transitioned to long term dialysis	7/38 (18.4%)	5/28 (17.9%)	2/10 (20.0%)	
Died on CRRT	20/38 (52.6%)	15/28 (53.6%)	5/10 (50.0%)	

*P values were not calculated for sub-cohorts given the very small sample size.*

CRRT, continuous renal replacement therapy; RRT, renal replacement therapy.

Six patients in the advanced kidney disease cohort were on RRT prior to implantation and were not included, as their RRT requirement could not be attributed to the implantation process.

## Discussion

The early mortality seen in our population of advanced kidney disease patients is not surprising given their demographics are consistent with a more decompensated phenotype. This is most clearly identified by lower INTERMACS profiles, although it should also be noted that lower plasma hemoglobin and bilirubin levels also suggest poorer oxygenation and increased congestion, respectively. Given these findings, the data showing similar survival in the long term is even more remarkable and is in line with previous research.^4^

It should also be noted that slightly more than half of patients with new CRRT requirements after implantation (regardless of preoperative renal function) died while on CRRT therapy. Clinicians should make note when counseling patients and families on CRRT after implantation that this is a strong negative prognostic indicator.

There is appropriate concern among clinicians that patients with advanced kidney disease may not tolerate LVAD implantation without being pushed into complete renal failure. Our data shows a trend toward higher rates of renal failure but is not large enough to detect a significant difference. It should be noted that the majority of our advanced kidney disease patients do not require renal replacement after LVAD implantation.

## Conclusion

It cannot be overstated that our dataset is limited in size, and interpretation should be made with caution, but we find that our selected population of patients with a GFR less than 30 achieve 2-year survival rates similar to controls. Post-implantation renal failure seems to occur at a higher rate, but a significant difference cannot be detected in our sample size. In the correct clinical context, LVAD implantation may be considered in patients with advanced kidney disease.

## Disclosure statement

The authors have no conflicts of interest to disclose.

## Financial support

This research did not receive any specific grant from funding agencies in the public, commercial, or not-for-profit sectors.

## References

[1] Yuzefpolskaya M., Schroeder S.E., Houston B.A. (2023). The Society of Thoracic Surgeons Intermacs 2022 Annual Report: focus on the 2018 heart transplant allocation system. Ann Thorac Surg.

[2] Mehra M.R., Goldstein D.J., Uriel N. (2018). Two-year outcomes with a magnetically levitated cardiac pump in heart failure. N Engl J Med.

[3] Lakhdar S., Nassar M., Buttar C. (2022). Outcomes with left ventricular assist device in end-stage renal disease: a systematic review. Cureus.

[4] Grinstein J., Kadakkal A., Rodrigo M. (2019). Advanced kidney disease in the left ventricular assist device population: impact on disease progression, morbidity and mortality. J Heart Lung Transpl.

[5] Levey A.S., Stevens L.A., Schmid C.H. (2009). A new equation to estimate glomerular filtration rate. Ann Intern Med.

[6] Therneau TM. A Package for Survival Analysis in R. 2023. Available at: https://CRAN.R-project.org/package=survival.

[7] Sjoberg D, Baillie M, Fruechtenicht C, Haesendonckx S. ggsurvfit: Flexibile Time-to-Event Figures. 2023; Published online 2024. https://github.com/pharmaverse/ggsurvfit.

[8] Wickham H. ggplot2: Elegant Graphics for Data Analysis. Published online 2016. https://ggplot2.tidyverse.org.

